# Supplementary material for: Methylated Reprimo Cell-Free DNA as a Non-Invasive Biomarker for Gastric Cancer
Source: Int J Mol Sci. 2025 Apr 3;26(7):3333. doi: 10.3390/ijms26073333 (PMC11989948; doi:10.3390/ijms26073333)
Supplement: Supplementary file 1 [file ijms-26-03333-s001.zip › Supplementary Figures.zip!/Supplementary Figures/Figure S2.pdf]

A

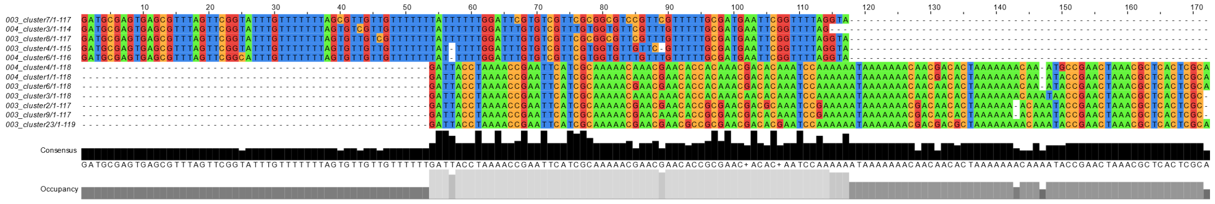

B

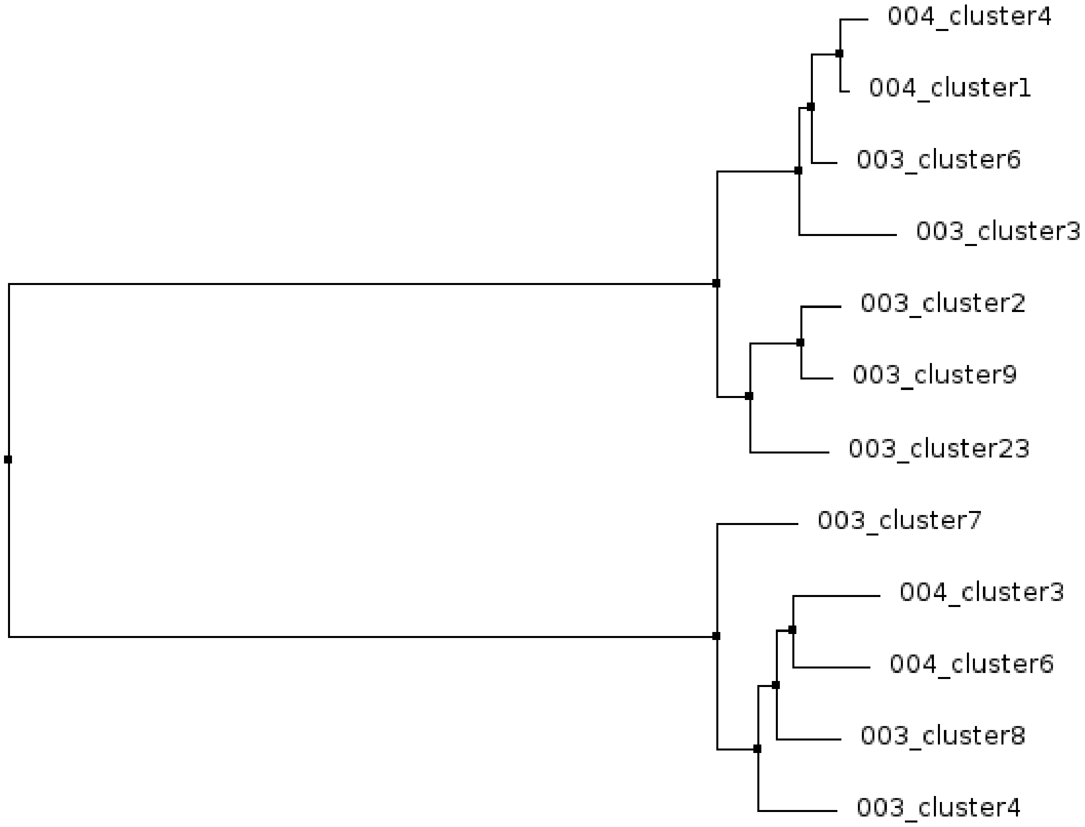

Figure S2 Targeted sequencing of PCR amplicon products from paired tumor and plasma samples from a gastric cancer case. A. Alignment of tissue samples (clusters 003) and plasma samples (clusters 004) by clustalW. B. Distance trees of the same tissue and plasma clusters by makeTree.py script. Four out of 8 clusters were found in tumor and plasma samples (003_cluster6 and 004_cluster1 & 4 and 003_cluster8 and 004_cluster3 &6).
