# Supplementary material for: Methylated Reprimo Cell-Free DNA as a Non-Invasive Biomarker for Gastric Cancer
Source: Int J Mol Sci. 2025 Apr 3;26(7):3333. doi: 10.3390/ijms26073333 (PMC11989948; doi:10.3390/ijms26073333)
Supplement: Supplementary file 1 [file ijms-26-03333-s001.zip › Supplementary Figures.zip!/Supplementary Figures/Figure S3.pdf]

A

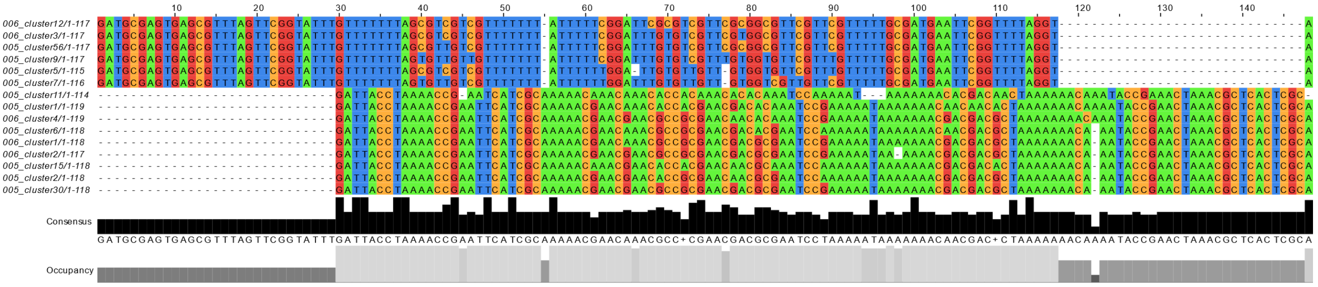

B

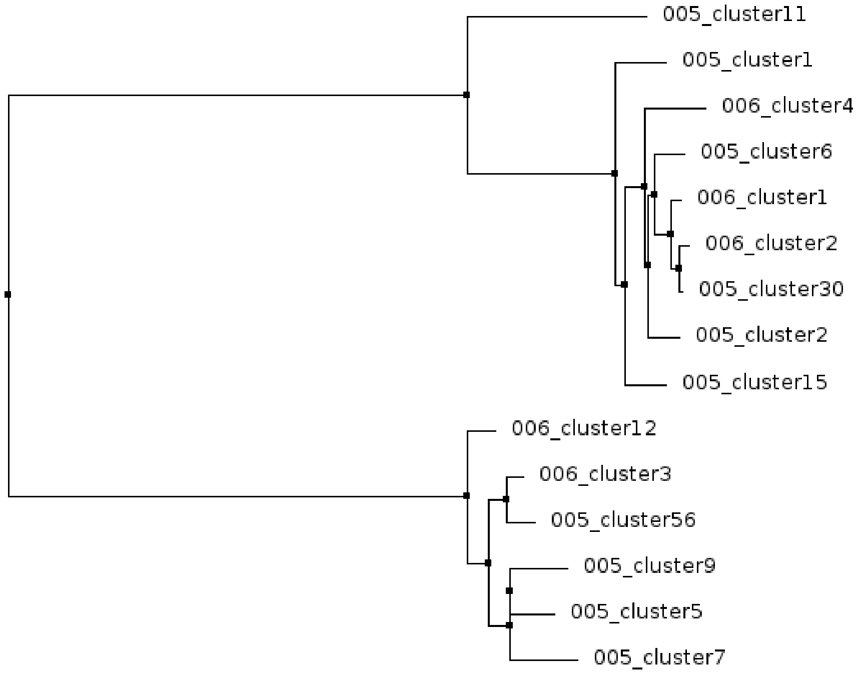

Figure S3 Targeted sequencing of PCR amplicon products from paired tumor and plasma samples from a gastric cancer case. A. Alignment of tissue samples (clusters 005) and plasma samples (clusters 006) by clustalW. B. Distance trees of the same tissue and plasma clusters by makeTree.py script. Five out of 10 clusters were found in tumor and plasma samples (005_cluster30 and 006_cluster1 & 2; 005_cluster56 and 006_cluster3; 005_cluster2 and 006_cluster4 and 005_cluster5, 7 &9 and 006_cluster12).
